# Dietary Salt Intake and Blood Pressure in a Representative Japanese Population: Baseline Analyses of NIPPON DATA80

**DOI:** 10.2188/jea.JE20090220

**Published:** 2010-05-05

**Authors:** Katsuyuki Miura, Nagako Okuda, Tanvir Chowdhury Turin, Naoyuki Takashima, Hideaki Nakagawa, Koshi Nakamura, Katsushi Yoshita, Akira Okayama, Hirotsugu Ueshima

**Affiliations:** 1 Department of Health Science, Shiga University of Medical Science, Ohtsu, Japan; 2 Department of Epidemiology and Public Health, Kanazawa Medical University, Ishikawa, Japan; 3 Nutritional Epidemiology Program, National Institute of Health and Nutrition, Tokyo, Japan; 4 First Institute of Health Service, Japan Anti-Tuberculosis Association, Tokyo, Japan

**Keywords:** salt intake, nutrient intake, food intake, blood pressure

## Abstract

**Background:**

The relationship between dietary salt intake and blood pressure (BP) has been rarely investigated in a large population of Japanese. The characteristics of nutrients intake and foods intake in Japanese people with high salt intake have also not investigated well.

**Methods:**

Data of 10 422 participants (4585 men and 5837 women) aged 30 or older who participated in both the National Survey on Circulatory Disorders and National Nutrition Survey in Japan conducted in 1980 were used. The nutrition surveys were performed with weighing record method for three consecutive days to each household. BP and intakes of nutrients and foods were compared by the quintiles of estimated individual salt intake per day. Analyses of covariance were used to calculate multivariate-adjusted mean BP values by the quintiles.

**Results:**

Participants with higher salt intake showed higher intakes of soy beans/legume, fruit, other vegetables, and fish/shellfish. Intakes of protein, potassium, calcium, iron, magnesium, and fiber were higher in higher quintiles of salt intake. In men, adjusted systolic BPs were higher in the higher salt intake quintiles; there was 4.3 mm Hg difference in multivariate-adjusted systolic BP between the lowest quintile (mean salt intake 8.7 g/day) and the highest quintile (mean salt intake 23.5 g/day) (*P* < 0.001). In women, adjusted mean systolic BPs were not statistically different among the quintile of salt intake.

**Conclusions:**

A positive relationship of dietary salt intake to BP was observed, especially in men, in this large-scale representative Japanese population.

The causal relationship between high salt (sodium chloride) intake and high blood pressure (BP) is now established worldwide. Evidence includes results from animal studies, epidemiological studies, clinical trials, and meta-analysis of trials. A salt-reduced diet is an established method to prevent and treat hypertension, and has been recommended in several guidelines for the treatment and prevention of hypertension.^1^^–^^5^

Japan has been one of the countries with high salt intake. In 1960, Dahl reported an ecological study on the relationship between salt intake and BP in various populations in the world, and, in this report, people living in northern Japan consumed about 30 g of salt per day and had the highest prevalence of hypertension.^6^ The main reason of a dramatic decrease in population-wide BP level in the past several decades in Japan is considered to be a marked decrease in salt intake in the whole Japanese population. However, the intra-population relationship between dietary salt intake and BP in Japan has been rarely investigated, mainly because high quality assessment of the amount of dietary salt intake is difficult in a large-scale epidemiological study.

The National Nutritional Survey of Japan (NNSJ) was initiated in 1946, and, recently, the survey has been conducted once every year.^7^ This survey has been performed using weighing record method for three consecutive days to each household. From this survey, high quality data on dietary salt intake are available in a large-scale sample of representative Japanese from 300 randomly selected districts in Japan. The majority of the participants for NNSJ also participated in the National Survey on Circulatory Disorders conducted every 10 years. Cohort studies based on the National Survey on Circulatory Disorders in 1980 and 1990 were names as the National Integrated Project for Prospective Observation of Non-communicable Disease and Its Trends in the Aged (NIPPON DATA80 and NIPPON DATA90).^8^^,^^9^

In the present report, we investigated the relationship of dietary salt intake to BP and to intakes of nutrients and foods in a large-scale representative Japanese using the baseline data of NIPPON DATA80, where high-quality data on dietary salt intake from NNSJ are available.

## METHODS

### Participants

The participants in this cohort were those in the 1980 National Survey on Circulatory Disorders.^10^ A total of 10 546 community-based participants aged 30 years and over in 300 randomly selected health districts throughout Japan participated in the survey, which consisted of history-taking, physical examinations, blood tests and self-administered questionnaires on lifestyle. Overall recruited population aged 30 years and over in the 300 participating health districts was 13 771; therefore, the participation rate of the survey was 76.6%.

### Baseline examination

At baseline, non-fasting blood samples were obtained. The serum was separated and centrifuged soon after blood coagulation. These samples were shipped to one labolatory (SRL, Tokyo) for blood chemistry measurements. Serum total cholesterol was measured enzymatically. Body mass index (BMI) was calculated as weight (kg) divided by the square of height (m). Baseline BP was measured by trained observers using a standard mercury sphygmomanometer on the right arm of seated participants. History of hypertension was asked whether participants were told as having hypertension by health professionals in the past. Participants were also asked whether they are treated by antihypertensive medicine at present.

### Nutritional survey

Detailed methods of the nutritional survey and the estimation of individual intake of nutrients and food groups were described elsewhere.^7^^,^^11^ Food intake survey by weighed food records in three consecutive representative days were conducted by specially trained dietary interviewers. Dietary interviewers visited participants’ houses at least once during the survey. Weekends and holydays were avoided. Nutrient intakes were calculated using Standard Tables for Food Composition in Japan, 3rd revised edition, were used for NNSJ80. Detailed nutrient intakes; e.g. fatty acid, cholesterol, etc., were calculated using representative nutrient compositions for food groups utilizing results from dietary survey in Japan conducted for an international cooperative epidemiological study.^11^

Nutrient intakes of each household member were estimated by dividing household intake data of NNSJ80 conducted in 1980 proportionally using average intakes by sex and age groups calculated for NNSJ95 conducted in 1995.^12^ The average intakes in NNSJ95 were calculated by a combination method of household-based food weighing record and an approximation of proportions by which family members shared each dish or food in the household. For each person, means of the estimated individual nutrients from the three days records were used in the analyses.

### Statistical analyses

The number of participants for analysis was 10 422 (4585 men and 5837 women) who had complete data. Analyses were done in men and in women separately. Characteristics and the intakes of various nutrients and food groups were compared by the quintiles of dietary salt intake (g/day). *P* for trend of mean values by the quintiles was calculated to examine a linear relationship, using the median of each quintile. Adjusted mean values of BP by the quintiles were calculated by the analysis of covariance, and multiple comparison tests were done compared with the lowest quintile of salt intake. Mean BP (systolic and diastolic) values were adjusted for age (model 1); for age, BMI (kg/m^2^), current drinking, potassium intake (mg/day), and total energy intake (kcal/day) (model 2); and for age, BMI (kg/m^2^), current drinking, total energy intake (kcal/day), vegetable intake (the sum of green and yellow vegetable and other vegetable) (g/day), fruit intake (g/day) and fish/shellfish intake (g/day) (model 3). The analyses were done in all participants and in participants without history of hypertension and antihypertensive treatment.

## RESULTS

### Characteristics by the quintiles of salt intake

Mean values or proportion of each characteristic by the quintile of dietary salt intake (g/day) are shown in Table 1 for men and in Table 2 for women. In men, the mean salt intake was 8.7 g/day in the lowest quintile, 14.3 g/day in the middle quintile and 23.5 g/day in the highest quintile. Mean age and height were not different among the quintiles, but mean weight and BMI were significantly higher in higher quintiles. Participants with higher salt intake were significantly higher in drinking rate, systolic BP (SBP), and diastolic BP (DBP). SBP in the highest quintile was 2.9 mm Hg higher than that in the lowest quintile; DBP was 1.8 mm Hg higher.

**Table 1.** Characteristics and the intake of nutrients and food groups according to the quintiles of salt intake in men: NIPPON DATA80

	Quintiles of salt intake (range [g/day])										*P* for trend

	<10.8		10.8–13.1		13.1–15.5		15.5–18.8		18.8≤		
Number of participants	917		917		917		917		917		
Salt intake (g/day)	8.7	(1.8)	12.0	(0.7)	14.3	(0.7)	17.0	(1.0)	23.5	(4.9)	
Age (year)	50.6	(14.9)	49.9	(13.7)	50.0	(13.3)	49.7	(12.8)	49.6	(12.0)	0.155
Height (cm)	162.3	(6.7)	162.4	(7.0)	162.5	(6.5)	162.1	(6.7)	161.6	(6.6)	0.554
Weight (kg)	58.4	(9.3)	59.1	(9.1)	59.5	(9.1)	59.7	(9.2)	59.9	(9.2)	<0.001
Body mass index (kg/m^2^)	22.1	(3.0)	22.3	(2.8)	22.5	(2.9)	22.7	(2.8)	22.9	(2.8)	<0.001
Current smoking (%)	61.5		64.7		62.1		61.2		65.7		0.172^a^
Current drinking (%)	68.7		74.6		74.8		75.8		77.3		<0.001^a^
Systolic blood pressure (mm Hg)	137.1	(21.6)	137.5	(21.6)	137.6	(20.8)	139.5	(21.0)	140.0	(20.7)	0.021
Diastolic blood pressure (mm Hg)	82.6	(12.4)	83.2	(12.7)	83.0	(12.4)	84.3	(12.5)	84.4	(12.1)	0.010
Serum total cholesterol (mg/dl)	188.3	(32.3)	187.9	(32.9)	186.8	(32.7)	186.3	(32.1)	182.1	(34.4)	0.143
Blood glucose (mg/dl)	132.6	(38.9)	128.1	(34.3)	128.9	(31.1)	132.3	(44.2)	133.2	(38.6)	0.991
History of hypertension (%)	20.5		19.2		21.1		21.7		22.2		0.560^a^
Antihypertensive treatment (%)	14.3		11.0		13.5		13.4		13.8		0.351^a^
Nutrient intake											
Total energy (kcal)	2072	(438)	2263	(385)	2415	(402)	2513	(439)	2743	(557)	<0.001
Carbohydrate (%kcal)	60.3	(7.0)	60.0	(6.2)	59.4	(6.3)	59.6	(6.1)	59.4	(6.6)	0.003
Protein (%kcal)	14.4	(2.3)	14.9	(1.8)	15.1	(1.9)	15.2	(2.0)	15.7	(2.3)	<0.001
Fat (%kcal)	20.1	(5.4)	19.9	(5.0)	20.3	(5.1)	20.0	(5.0)	19.6	(5.5)	0.914
Animal protein (%kcal)	8.5	(2.9)	8.6	(2.3)	8.7	(2.2)	8.7	(2.3)	8.8	(2.6)	0.041
Vegetable protein (%kcal)	6.9	(1.0)	7.2	(0.9)	7.2	(0.9)	7.4	(0.9)	7.6	(1.0)	<0.001
Saturated fatty acids (%kcal)	5.8	(1.6)	5.7	(1.4)	5.8	(1.5)	5.6	(1.4)	5.4	(1.5)	0.006
Monounsaturated fatty acids (%kcal)	7.6	(2.0)	7.4	(1.9)	7.6	(1.9)	7.4	(1.9)	7.2	(2.1)	0.234
Polyunsaturated fatty acids (%kcal)	5.2	(1.3)	5.2	(1.3)	5.3	(1.3)	5.3	(1.4)	5.4	(1.6)	0.046
n-3 fatty acid (%kcal)	1.0	(0.3)	1.1	(0.3)	1.1	(0.3)	1.1	(0.3)	1.2	(0.4)	<0.001
Dietary cholesterol (mg/1000 kcal)	164	(68)	166	(54)	167	(53)	163	(52)	164	(54)	0.957
Potassium (mg/1000 kcal)	1168	(229)	1252	(220)	1269	(228)	1301	(227)	1381	(243)	<0.001
Calcium (mg/1000 kcal)	211	(62)	225	(58)	228	(58)	234	(58)	250	(59)	<0.001
Iron (mg/1000 kcal)	5.6	(1.0)	6.1	(1.0)	6.2	(1.0)	6.5	(1.1)	7.1	(1.2)	<0.001
Phosphorus (mg/1000 kcal)	564	(80)	575	(63)	580	(64)	586	(66)	601	(72)	<0.001
Magnesium (mg/1000 kcal)	126	(18)	134	(17)	137	(18)	141	(19)	149	(21)	<0.001
Vitamin A (IU/1000 kcal)	697	(315)	748	(316)	742	(317)	717	(313)	727	(318)	0.248
Vitamin B1 (mg/1000 kcal)	0.48	(0.17)	0.47	(0.16)	0.48	(0.17)	0.49	(0.16)	0.48	(0.15)	0.125
Vitamin B2 (mg/1000 kcal)	0.38	(0.11)	0.40	(0.10)	0.41	(0.10)	0.41	(0.10)	0.42	(0.10)	<0.001
Vitamin C (mg/1000 kcal)	43.1	(19.3)	47.1	(18.4)	47.3	(18.0)	46.2	(17.2)	47.9	(18.1)	<0.001
Fiber (g/1000 kcal)	6.9	(1.7)	7.5	(1.6)	7.6	(1.7)	7.8	(1.8)	8.3	(1.8)	<0.001
Food intake											
Cereals (g/1000 kcal)	170.3	(32.5)	163.2	(30.3)	159.0	(28.8)	156.7	(27.7)	151.6	(30.4)	<0.001
Rice (g/1000 kcal)	130.6	(36.5)	125.9	(34.5)	124.7	(35.3)	125.5	(32.2)	125.2	(34.3)	0.001
Flour (g/1000 kcal)	41.5	(29.4)	39.4	(25.5)	36.9	(23.8)	34.3	(22.9)	30.0	(21.8)	<0.001
Nuts (g/1000 kcal)	0.4	(1.5)	0.5	(1.5)	0.5	(1.4)	0.6	(1.7)	0.6	(2.4)	0.042
Potatoes (g/1000 kcal)	23.5	(18.7)	28.7	(20.4)	28.2	(18.4)	28.1	(17.8)	29.9	(20.4)	<0.001
Sugar/sweetener (g/1000 kcal)	5.1	(3.7)	5.7	(4.1)	5.7	(3.6)	6.0	(4.0)	6.1	(4.8)	<0.001
Sweet/snacks (g/1000 kcal)	5.9	(7.7)	6.1	(6.8)	6.4	(7.2)	6.8	(6.7)	6.5	(7.5)	0.005
Fats/oils (g/1000 kcal)	7.3	(4.1)	7.0	(4.2)	7.5	(4.4)	7.0	(4.2)	6.8	(4.9)	0.567
Soy beans/legume (g/1000 kcal)	29.2	(21.1)	33.8	(22.5)	34.7	(20.0)	35.7	(19.5)	40.1	(21.0)	<0.001
Fruit (g/1000 kcal)	52.5	(41.2)	59.7	(39.5)	62.1	(39.2)	60.0	(39.0)	61.0	(39.7)	<0.001
Green and yellow vegetables (g/1000 kcal)	23.1	(17.0)	24.1	(16.0)	24.4	(16.8)	23.5	(16.0)	24.0	(17.0)	0.547
Other vegetables (g/1000 kcal)	83.0	(33.5)	91.3	(36.6)	92.7	(33.6)	100.8	(38.1)	115.5	(45.7)	<0.001
Mushrooms (g/1000 kcal)	3.8	(5.2)	3.8	(5.3)	3.9	(4.9)	4.7	(5.9)	4.5	(6.0)	<0.001
Sea algae (g/1000 kcal)	1.8	(2.0)	2.3	(3.0)	2.7	(3.4)	3.0	(3.7)	3.6	(4.4)	<0.001
Condiment/beverage (g/1000 kcal)	74.8	(95.2)	77.9	(67.8)	81.0	(68.7)	85.2	(70.2)	92.5	(69.9)	0.002
Fish/shellfish (g/1000 kcal)	47.2	(24.1)	50.3	(24.3)	51.4	(24.0)	53.7	(23.6)	58.9	(29.7)	<0.001
Meat (g/1000 kcal)	31.6	(19.3)	29.7	(15.5)	30.7	(16.4)	29.5	(15.3)	26.0	(15.1)	0.026
Egg (g/1000 kcal)	17.6	(11.4)	18.0	(9.5)	17.8	(9.2)	16.4	(9.0)	16.0	(8.5)	0.008
Milk/dairy products (g/1000 kcal)	35.0	(30.3)	32.3	(24.3)	31.3	(24.5)	28.0	(21.7)	25.1	(22.3)	<0.001
Other foods (g/1000 kcal)	2.9	(9.9)	2.7	(5.9)	2.9	(7.0)	2.8	(5.6)	2.8	(5.5)	0.998

Values are mean (standard deviation) or proportion (%).

^a^*P* values by chi-square test.

**Table 2.** Characteristics and the intake of nutrients and food groups according to the quintiles of salt intake in women: NIPPON DATA80

	Quintiles of salt intake (range [g/day])										*P* for trend
	
	<9.2		9.2–11.2		11.2–13.3		13.3–16.2		16.2≤		
Number of participants	1167		1168		1167		1168		1167		
Salt intake (g/day)	7.6	(1.4)	10.2	(0.6)	12.2	(0.6)	14.6	(0.8)	20.2	(4.1)	
Age (year)	50.5	(14.9)	49.4	(13.9)	49.3	(13.0)	49.4	(12.9)	51.6	(12.4)	0.043
Height (cm)	149.9	(6.3)	150.4	(6.4)	150.5	(5.9)	150.2	(6.2)	149.5	(6.1)	0.029
Weight (kg)	50.4	(8.6)	51.7	(8.4)	51.2	(7.9)	51.7	(8.2)	52.2	(8.3)	0.004
Body mass index (kg/m^2^)	22.4	(3.4)	22.9	(3.5)	22.6	(3.3)	22.9	(3.3)	23.4	(3.3)	0.011
Current smoking (%)	13.0		8.8		10.1		7.8		9.3		<0.001^a^
Current drinking (%)	19.2		22.6		22.4		18.8		18.6		0.022^a^
Systolic blood pressure (mm Hg)	133.7	(23.1)	133.7	(22.8)	132.7	(21.3)	133.9	(21.3)	134.9	(21.1)	0.894
Diastolic blood pressure (mm Hg)	79.1	(12.6)	79.2	(12.3)	79.1	(12.0)	79.5	(11.4)	80.7	(12.3)	0.413
Serum total cholesterol (mg/dl)	193.3	(34.2)	192.0	(34.9)	190.4	(34.8)	191.0	(33.1)	188.7	(34.4)	0.066
Blood glucose (mg/dl)	127.6	(37.3)	128.8	(35.2)	127.7	(28.9)	129.8	(31.3)	131.9	(35.3)	0.223
History of hypertension (%)	19.7		20.9		18.0		19.9		22.2		0.177^a^
Antihypertensive treatment (%)	14.6		15.0		13.1		14.6		16.4		0.331^a^
Nutrient intake											
Total energy (kcal)	1673	(348)	1823	(314)	1934	(325)	2020	(348)	2198	(454)	<0.001
Carbohydrate (%kcal)	62.6	(7.3)	62.0	(6.5)	61.7	(6.9)	62.0	(6.4)	62.1	(6.9)	0.017
Protein (%kcal)	14.8	(2.1)	15.3	(1.9)	15.5	(1.9)	15.6	(2.1)	16.2	(2.2)	<0.001
Fat (%kcal)	21.9	(6.1)	21.9	(5.5)	22.1	(5.8)	21.7	(5.6)	21.0	(5.9)	0.782
Animal protein (%kcal)	8.7	(2.8)	8.9	(2.3)	8.9	(2.4)	8.9	(2.4)	9.0	(2.7)	0.022
Vegetable protein (%kcal)	7.2	(1.0)	7.3	(0.9)	7.4	(1.0)	7.6	(0.9)	7.9	(1.1)	<0.001
Saturated fatty acids (%kcal)	6.4	(1.8)	6.3	(1.6)	6.3	(1.7)	6.1	(1.6)	5.8	(1.6)	0.005
Monounsaturated fatty acids (%kcal)	8.2	(2.3)	8.2	(2.0)	8.3	(2.2)	8.1	(2.1)	7.8	(2.3)	0.239
Polyunsaturated fatty acids (%kcal)	5.6	(1.5)	5.7	(1.4)	5.8	(1.5)	5.8	(1.5)	5.8	(1.6)	0.006
n-3 fatty acid (%kcal)	1.1	(0.4)	1.2	(0.4)	1.2	(0.4)	1.2	(0.4)	1.3	(0.4)	<0.001
Dietary cholesterol (mg/1000 kcal)	178	(69)	180	(56)	180	(56)	178	(59)	178	(62)	0.955
Potassium (mg/1000 kcal)	1328	(266)	1398	(244)	1437	(262)	1471	(260)	1579	(294)	<0.001
Calcium (mg/1000 kcal)	253	(68)	268	(67)	275	(65)	281	(68)	302	(73)	<0.001
Iron (mg/1000 kcal)	6.2	(1.1)	6.7	(1.1)	6.9	(1.1)	7.3	(1.2)	8.0	(1.4)	<0.001
Phosphorus (mg/1000 kcal)	588	(75)	599	(68)	605	(67)	610	(71)	629	(76)	<0.001
Magnesium (mg/1000 kcal)	135	(20)	141	(19)	146	(20)	149	(21)	160	(24)	<0.001
Vitamin A (IU/1000 kcal)	838	(410)	861	(356)	866	(372)	852	(365)	867	(397)	0.335
Vitamin B1 (mg/1000 kcal)	0.59	(0.20)	0.59	(0.19)	0.61	(0.21)	0.60	(0.19)	0.59	(0.19)	0.041
Vitamin B2 (mg/1000 kcal)	0.56	(0.16)	0.56	(0.15)	0.57	(0.15)	0.56	(0.14)	0.59	(0.15)	0.046
Vitamin C (mg/1000 kcal)	57.7	(26.0)	60.4	(22.8)	62.1	(23.4)	61.3	(22.7)	65.0	(25.0)	<0.001
Fiber (g/1000 kcal)	8.4	(2.0)	8.9	(1.8)	9.2	(1.9)	9.4	(2.0)	10.1	(2.2)	<0.001
Food intake											
Cereals (g/1000 kcal)	164.0	(30.9)	157.4	(29.8)	153.5	(28.6)	151.7	(26.8)	145.8	(29.4)	<0.001
Rice (g/1000 kcal)	113.9	(35.3)	108.6	(32.8)	107.0	(32.9)	107.2	(30.6)	109.0	(32.0)	<0.001
Flour (g/1000 kcal)	49.1	(31.7)	47.2	(29.9)	45.0	(28.5)	42.7	(27.0)	35.0	(25.2)	<0.001
Nuts (g/1000 kcal)	0.7	(2.4)	0.8	(2.6)	0.8	(2.3)	0.9	(2.8)	0.9	(2.7)	0.047
Potatoes (g/1000 kcal)	30.0	(23.0)	32.9	(22.7)	33.5	(21.7)	34.5	(23.0)	36.7	(26.2)	<0.001
Sugar/sweetener (g/1000 kcal)	6.2	(4.5)	6.5	(4.7)	6.7	(4.4)	7.1	(4.7)	7.4	(5.5)	<0.001
Sweet/snacks (g/1000 kcal)	11.9	(13.5)	12.8	(12.6)	13.5	(13.4)	14.6	(13.9)	13.9	(14.8)	<0.001
Fats/oils (g/1000 kcal)	8.1	(4.6)	8.0	(4.5)	8.3	(4.8)	7.9	(4.8)	7.4	(5.1)	0.712
Soy beans/legume (g/1000 kcal)	32.3	(23.4)	35.6	(23.0)	37.0	(21.5)	38.6	(21.1)	43.3	(22.5)	<0.001
Fruit (g/1000 kcal)	85.0	(60.4)	93.5	(54.6)	101.1	(59.3)	98.4	(59.3)	102.4	(65.9)	<0.001
Green and yellow vegetables (g/1000 kcal)	30.4	(22.9)	30.2	(19.4)	30.7	(20.9)	30.6	(20.6)	31.5	(23.2)	0.713
Other vegetables (g/1000 kcal)	94.8	(39.3)	102.5	(39.8)	105.8	(40.2)	112.0	(41.7)	132.5	(50.5)	<0.001
Mushrooms (g/1000 kcal)	4.1	(5.7)	4.3	(5.9)	4.6	(5.7)	5.2	(6.4)	5.2	(6.6)	<0.001
Sea algae (g/1000 kcal)	2.1	(2.7)	2.7	(3.4)	3.1	(3.9)	3.7	(4.7)	4.3	(5.4)	<0.001
Condiment/beverage (g/1000 kcal)	31.4	(56.5)	33.7	(33.1)	35.5	(30.9)	35.5	(31.9)	39.7	(43.4)	0.008
Fish/shellfish (g/1000 kcal)	45.5	(23.4)	48.5	(23.6)	49.4	(23.3)	51.4	(23.2)	56.9	(27.7)	<0.001
Meat (g/1000 kcal)	29.3	(16.9)	28.7	(15.2)	28.9	(16.1)	27.5	(14.7)	25.1	(16.3)	0.016
Egg (g/1000 kcal)	19.2	(12.2)	19.1	(9.7)	18.7	(9.4)	17.9	(9.9)	17.0	(9.9)	0.001
Milk/dairy products (g/1000 kcal)	52.7	(41.1)	50.9	(37.5)	49.0	(35.5)	45.7	(33.6)	38.8	(34.3)	<0.001
Other foods (g/1000 kcal)	3.0	(8.2)	3.3	(7.3)	3.1	(6.6)	3.2	(6.6)	3.2	(6.3)	0.603

Values are mean (standard deviation) or proportion (%).

^a^*P* values by chi-square test.

In women, the mean salt intake was 7.6 g/day in the lowest quintile, 12.2 g/day in the middle quintile and 20.2 g/day in the highest quintile, which were 1.1 to 3.3 grams lower than men. Mean age, height, weight, and BMI were higher in higher quintiles of salt intake (Table 2). Mean SBP and DBP were not different among the quintiles.

### Nutrients and foods intake by the quintiles of salt intake

In men, total energy intake was significantly higher in higher quintiles of salt intake (Table 1). Intakes of protein (%kcal), potassium (mg/1000 kcal), calcium (mg/1000 kcal), iron (mg/1000 kcal), phosphorus (mg/1000 kcal), magnesium (mg/1000 kcal), vitamin B2 and C (mg/1000 kcal), and fiber (g/1000 kcal) were higher in higher quintiles. Total fat intake (%kcal) was not different among the quintiles. Results for these nutrients were similar in women (Table 2).

Men with higher salt intake showed higher intakes (g/1000 kcal) of potatoes, sugar/sweetener, soy beans/legume, fruit, other vegetables, sea algae, and fish/shellfish, and showed lower intakes (g/1000 kcal) of cereals, flour, meat, and milk/dairy products (Table 1). Results for these food groups were similar in women (Table 2).

### Salt intake and BP

Table 3 shows adjusted mean values of SBP by the quintile of salt intake (g/day) in men and women. In all men, age-adjusted mean SBP tended to increase as salt intake increases (model 1). Age-adjusted SBP in the 4th and the highest quintile of salt intake were significantly higher than that in the lowest quintile; 3.0 mm Hg higher in the 4th quintile and 3.5 mm Hg higher in the highest quintile. Results were similar after adjustment of other confounders including BMI, current drinking, potassium intake, and total energy intake (model 2); adjusted SBP in the highest quintile was 4.3 mm Hg higher than that in the lowest quintile (*P* < 0.001). Results were also similar after adjustment of intakes of food groups (vegetable, fruit, and fish/shellfish) (model 3). When analyzing in men without history of hypertension and antihypertensive treatment, the above tendency was remained; multivariate-adjusted SBP in the highest quintile was 3.6 mm Hg higher than that in the lowest quintile (*P* < 0.001) (model 2).

**Table 3.** Adjusted mean values of systolic blood pressure by the quintiles of salt intake in men and women: NIPPON DATA80

	Quintiles of salt intake (g/day)								

	Q1 (lowest)	Q2		Q3		Q4		Q5 (highest)	
				
	Adjusted SBP (mm Hg)	Adjusted SBP (mm Hg)	*P*	Adjusted SBP (mm Hg)	*P*	Adjusted SBP (mm Hg)	*P*	Adjusted SBP (mm Hg)	*P*
Men, all (*n* = 4585)									
Model 1	136.7	137.5	0.365	137.6	0.300	139.7	0.001	140.2	<0.001
Model 2	136.5	137.4	0.307	137.6	0.232	139.6	<0.001	140.8	<0.001
Model 3	136.9	137.6	0.490	137.6	0.439	139.5	0.006	140.2	0.002
Men without history of hypertension and/or antihypertensive treatment (*n* = 3616)									
Model 1	131.5	131.7	0.819	132.4	0.316	133.6	0.014	135.2	<0.001
Model 2	131.6	131.7	0.969	132.3	0.442	133.5	0.041	135.2	<0.001
Model 3	132.0	131.8	0.870	132.3	0.623	133.4	0.109	134.8	0.005

Women, all (*n* = 5837)									
Model 1	133.3	134.2	0.292	133.3	0.933	134.5	0.157	133.6	0.704
Model 2	133.9	134.1	0.815	133.5	0.625	134.4	0.523	133.3	0.466
Model 3	134.0	134.2	0.863	133.6	0.562	134.4	0.682	133.1	0.318
Women without history of hypertension and/or antihypertensive treatment (*n* = 4765)									
Model 1	127.6	127.5	0.847	128.5	0.220	128.8	0.113	128.7	0.126
Model 2	127.9	127.6	0.673	128.5	0.375	128.7	0.286	128.3	0.618
Model 3	128.0	127.7	0.627	128.6	0.458	128.7	0.374	128.1	0.917

Mean values are adjusted by analysis of covariance. *P* values are compared with the lowest quintile by multiple comparison.

Model 1 is adjusted for age.

Model 2 is adjusted for age (years), body mass index (kg/m^2^), current drinking, potassium intake (mg/day), and total energy intake (kcal/day).

Model 3 is adjusted for age (years), body mass index (kg/m^2^), current drinking, total energy intake (kcal/day), vegetable intake (the sum of green, yellow vegetable and other vegetable) (g/day), fruit intake (g/day) and fish/shellfish intake (g/day).

SBP, systolic blood pressure.

In all women, adjusted mean values of SBP were not statistically different among the quintile of salt intake in any models (Table 3). Results were similar in women without history of hypertension; there were no significant differences in adjusted SBPs among the quintiles.

Table 4 shows adjusted mean values of DBP by the quintile of salt intake (g/day) in men and women. In all men, age-adjusted mean DBPs showed a tendency to increase as salt intake increases. Age-adjusted DBP in the highest quintile was 1.8 mm Hg higher than that in the lowest quintile (*P* < 0.001). The difference remained significant after adjustment of BMI, potassium intake, and total energy intake (1.3 mm Hg, *P* = 0.047) (model 2), although it was not significant after adjustment of intake of food groups (model 3). In men without history of hypertension, age-adjusted DBP in the highest quintile was 1.6 mm Hg higher than that in the lowest quintile (*P* = 0.006), but the difference was not significant after adjustment of confounders.

**Table 4.** Adjusted mean values of diastolic blood pressure by the quintiles of salt intake in men and women: NIPPON DATA80

	Quintiles of salt intake (g/day)								

	Q1 (lowest)	Q2		Q3		Q4		Q5 (highest)	
				
	Adjusted DBP (mm Hg)	Adjusted DBP (mm Hg)	*P*	Adjusted DBP (mm Hg)	*P*	Adjusted DBP (mm Hg)	*P*	Adjusted DBP (mm Hg)	*P*
Men, all (*n* = 4585)									
Model 1	82.6	83.3	0.231	83.0	0.455	84.3	0.002	84.4	0.001
Model 2	82.9	83.4	0.421	83.0	0.931	84.2	0.035	84.2	0.047
Model 3	83.2	83.5	0.612	83	0.751	84.1	0.137	83.8	0.362
Men without history of hypertension and/or antihypertensive treatment (*n* = 3616)									
Model 1	80.2	80.5	0.616	80.4	0.760	81.6	0.012	81.8	0.006
Model 2	80.7	80.6	0.937	80.4	0.596	81.5	0.188	81.4	0.242
Model 3	80.9	80.7	0.809	80.4	0.416	81.4	0.400	81.1	0.733

Women, all (*n* = 5837)									
Model 1	79.0	79.3	0.443	79.3	0.549	79.7	0.129	80.4	0.004
Model 2	79.7	79.4	0.536	79.4	0.547	79.6	0.746	79.7	0.950
Model 3	79.7	79.4	0.526	79.4	0.525	79.6	0.699	79.6	0.799
Women without history of hypertension and/or antihypertensive treatment (*n* = 4765)									
Model 1	76.6	76.3	0.511	77.0	0.465	77.4	0.101	78.0	0.005
Model 2	77.2	76.5	0.162	77	0.768	77.3	0.818	77.3	0.781
Model 3	77.2	76.5	0.181	77	0.807	77.3	0.750	77.3	0.750

Mean values are adjusted by analysis of covariance. *P* values are compared with the lowest quintile by multiple comparison.

Model 1 is adjusted for age.

Model 2 is adjusted for age (years), body mass index (kg/m^2^), current drinking, potassium intake (mg/day), and total energy intake (kcal/day).

Model 3 is adjusted for age (years), body mass index (kg/m^2^), current drinking, total energy intake (kcal/day), vegetable intake (the sum of green, yellow vegetable and other vegetable) (g/day), fruit intake (g/day) and fish/shellfish intake (g/day).

DBP, diastolic blood pressure.

In all women and women without history of hypertension, age-adjusted DBPs showed an increasing tendency as salt intake increases (Table 4). However, there were no significant differences in multivariate-adjusted DBPs among the quintile (model 2 and model 3).

When the energy density of salt intake (g/1000 kcal) was used instead of the absolute amount of salt intake (g/day) for the above analyses, results were similar.

## DISCUSSION

In the present report, we investigated the relationship of dietary salt intake to BP in a large-scale representative Japanese, in whom high-quality data on dietary salt intake from the National Nutrition Survey of Japan are available, and we found a significant independent relationship between the amount of salt intake and BP especially in men. There was 4.3 mm Hg difference in multivariate-adjusted SBP between the lowest quintile (mean salt intake 8.7 g/day) and the highest quintile (mean salt intake 23.5 g/day). This would be the first report demonstrating salt intake-BP relationship within a large Japanese population, where salt intake was assessed using weighing record method for three consecutive days in households.

A famous report by Dahl was an ecological study on the relationship between salt intake and BP in populations in various parts of the world, including Japan.^6^ However, the design of this study was not standardized for both of BP measurement and the assessment of salt intake. The international INTERSALT study was conducted, using 52 populations in 32 countries, with strictly standardized BP measurement and evaluation of salt intake by 24-hour urinary sodium excretion determination.^13^^–^^15^ The INTERSALT study found that urinary sodium excretion was significantly related to individual BP and to age-dependent BP elevation, after adjustment for age, gender, obesity and alcohol consumption. It was estimated that a decrease in sodium excretion by 100 mmol (equivalent to about 5.8 g salt) lowered SBP by 2.2 mm Hg, and that SBP and DBP were reduced by 10–11 mm Hg and 6 mm Hg, respectively, between 25 and 55 years of age—a period of 30 years. The measurement of 24-hour urinary sodium excretion is considered to be the gold standard to assess dietary salt intake. However, this method is usually difficult to perform in a large-scale epidemiological study. Our results assessing salt intake by 3-day weighing record method in the National Nutrition Survey were a valuable finding, and the magnitude of salt intake-SBP relation in men would be reasonable compared to the finding in the INTERSALT.

In women, we could not find a significant relationship between salt intake and BP after adjustment of confounders, even when excluding participants with history of hypertension or antihypertensive treatment. The National Survey of Circulatory Disorder in Japan showed that, in the 30 years from 1971 to 2000, mean SBP in Japanese substantially decreased both in men and women and in younger and older age groups.^16^^,^^17^ As the mean BP decreased in the whole population, including the younger generations, such a phenomenon is considered to be explained by a population-wide reduced consumption of salt through the nationwide campaign in this period.^18^ Our female participants in 1980 may have begun to change their dietary habit to reduce salt intake, which would cause a null relationship between salt intake and BP in this cross-sectional study design.

In the present study, we could clarify the characteristics of nutrient intake and dietary habits in Japanese people with high salt intake. For food groups, people with higher salt intake showed higher intakes of soy beans/legume, vegetables, and fish/shellfish, which means they have Japanese style dietary pattern. On the other hand, people with lower salt intake showed higher intakes of meat and milk/dairy products, which means they have relatively westernized dietary pattern. For nutrients, people with higher salt intake showed higher intakes of potassium, phosphorus, magnesium, and fiber, which would be mainly due to higher intake of vegetables. For the prevention of hypertension and cerebro-cardiovascular diseases, Japanese should reduce salt intake with keeping intakes of vegetable, fruit, soy product, and fish/shellfish high.

One of the limitations of this study is that the amount of alcohol consumption was not adjusted in multivariate-adjusted models. If participants with high salt intake tend to consume more alcohol, the salt-BP relationship would be overestimated especially in men. Another limitation is that physical activity was not considered in the models. It is possible that rural people consume more salt and are more physically active with labor; it may cause underestimation of the salt-BP relationship. A limitation of our method to estimate individual nutrient intake from dietary record in household has been described.^11^

In conclusion, a positive relationship of dietary salt intake to BP was observed, especially in men, in this large-scale representative Japanese population, in whom high-quality data on dietary salt intake from the National Nutrition Survey of Japan are available. The long-term mortality risk of cerebro-cardiovascular diseases should be investigated in relation to baseline salt intake in the NIPPON DATA cohorts.
